# Efficacy and safety of cardiac resynchronization therapy in chemotherapy‐induced cardiomyopathy: A systematic review

**DOI:** 10.1111/anec.13070

**Published:** 2023-07-12

**Authors:** Muhammad Shehram, Hiba Khalid, Hafiz Muhammad Shafique, Bakht Umer, Awais Zafar, Asif Ullah, Syed Muhammad Jawad Zaidi, Jawad Basit, Amin Mehmoodi, Jahanzeb Malik

**Affiliations:** ^1^ Department of Cardiology Mufti Mehmood Memorial Teaching Hospital DI Khan Pakistan; ^2^ Department of Dermatology Benazir Bhutto Hospital Rawalpindi Pakistan; ^3^ Department of Interventional Cardiology Armed Forces Institute of Cardiology Rawalpindi Pakistan; ^4^ Department of Medicine Sahiwal Medical College Sahiwal Pakistan; ^5^ Department of Cardiology Khyber Medical University Institute of Medical Sciences Kohat Pakistan; ^6^ Department of Cardiovascular Medicine Cardiovascular Analytics Group Canterbury UK; ^7^ Department of Medicine Ibn e Seen Hospital Kabul Afghanistan

**Keywords:** anthracycline, diastolic dysfunction, ejection fraction, heart failure

## Abstract

**Objective:**

The aim of the study was to assess the efficacy of cardiac resynchronization therapy (CRT) in patients with chemotherapy‐induced cardiomyopathy (CIC).

**Methods:**

With the increasing incidence of CIC, the association of CRT with improvement in clinical outcomes, echocardiographic parameters, and New York Heart Classification (NYHA) class was assessed through this qualitative systematic review.

**Results:**

The five studies included a total of 169 patients who underwent CRT after CIC, and of these, 61 (36.1%) patients were males. All studies showed an improvement in left ventricular ejection fraction (LVEF), among other echocardiographic parameters of LV volume. However, these findings are limited by short follow‐up periods, small sample sizes, and the absence of a control group.

**Conclusion:**

CRT was associated with improvement in all patient parameters with CIC.

## Graphical abstract

The five studies included a total of 169 patients who underwent CRT after CIC, which showed an improvement in left ventricular ejection fraction (LVEF), among other echocardiographic parameters of LV volume. However, these findings are limited by short follow‐up periods, small sample sizes, and the absence of a control group.

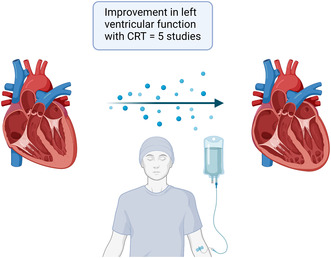

## 1 INTRODUCTION

Chemotherapy is a crucial treatment option for various types of cancer, however, its use is associated with several adverse effects, one of which is chemotherapy‐induced cardiomyopathy (CIC) (Ezzeddine et al., 2021; Fadol et al., 2017; Patel et al., 2022; Rickard et al., 2010; Singh et al., 2019). CIC is characterized by a decline in left ventricular function, leading to symptoms of heart failure (Rickard et al., 2010). The prevalence of CIC has increased in recent years due to the increasing use of chemotherapy in cancer patients (Singh et al., 2019). The management of CIC remains a challenge for healthcare providers, as conventional therapies for heart failure may not be effective in this patient population (Singh et al., 2019).

Cardiac resynchronization therapy (CRT) is a widely used treatment for heart failure in a wide range of etiologies (Singh et al., 2019). It involves the placement of a biventricular pacemaker that resynchronizes the electrical activity of the ventricles, leading to improved cardiac function (Ezzeddine et al., 2021). Despite the widespread use of CRT, there is limited information on its efficacy and safety in patients with CIC. Therefore, a systematic review of the existing literature is necessary to determine the role of CRT in the management of CIC.

The purpose of this systematic review is to summarize the available evidence on the efficacy and safety of CRT in patients with CIC. The review will provide a summary of the available evidence on using CRT in CIC and will help guide clinical decision‐making in this patient population. The findings of this review will also help in understanding the role of CRT in the management of CIC and provide a foundation for future research in this area. Additionally, this systematic review will help healthcare providers in making informed decisions regarding the use of CRT in patients with CIC. It will also help in developing evidence‐based guidelines for the management of this patient population. Overall, this review will contribute to advancing knowledge on the use of CRT in patients with CIC and will provide valuable information to healthcare providers and patients.

## 2 METHODS

### 2.1 Search strategy and selection criteria

The search strategy for the systematic review was as follows:

Electronic databases: The search was conducted in multiple electronic databases including PubMed, Embase, and the Cochrane Library.
Keywords: The search was performed using the following keywords and their combinations: "cardiac resynchronization therapy", "CRT", "chemotherapy‐induced cardiomyopathy", "CIC", "efficacy", and "safety".
Inclusion criteria: The inclusion criteria for the studies were as follows:
  - Studies evaluating the efficacy and safety of CRT in patients with CIC.
  - No time filters or language restrictions were placed on the search.
  - Randomized controlled trials, observational studies, and case–control studies.
Data extraction: The data from eligible studies were extracted and analyzed using a standardized data extraction form, including study design, sample size, patient demographics, and outcome measures.
Quality assessment: The quality of the included studies was assessed using the Cochrane Risk of Bias tool for randomized controlled trials (RCTs) and the Newcastle‐Ottawa Scale for observational studies.

Two independent reviewers screened the studies for eligibility based on the selection criteria. Any discrepancies were resolved through consensus.

### 2.2 Data extraction

The data extraction for the systematic review was performed as follows:

Data extraction form: A standardized data extraction form was used to extract data from eligible studies. The form included the following information:
  - Study design.
  - Sample size.
  - Patient demographics, including age, gender, and type of cancer.
  - Outcome measures, including left ventricular ejection fraction (LVEF), quality of life scores, and adverse events.
Data extraction process: Two independent reviewers extracted data from the eligible studies using the data extraction form. Any discrepancies were resolved through consensus.
Data analysis: The extracted data were analyzed to summarize the available evidence on the efficacy and safety of CRT in patients with CIC. The data was analyzed for the following outcomes:
  - Improvement in LVEF.
  - Quality of life scores.
  - Adverse events, including arrhythmias, device‐related infections, and lead dislodgement.
Data synthesis: The extracted data was synthesized and analyzed to provide a comprehensive summary of the available evidence on the efficacy and safety of CRT in patients with CIC. The results of the studies were compared and contrasted to determine the overall effectiveness of CRT in this patient population.

### 2.3 Statistical analysis

All data were processed in the Statistical Package for Social Sciences (SPSS) version 26 (IBM Corp.). The continuous data were presented as mean and standard deviation (SD) while categorical data were presented as frequency and percentages.

## 3 RESULTS

### 3.1 Search results

**FIGURE 1:**
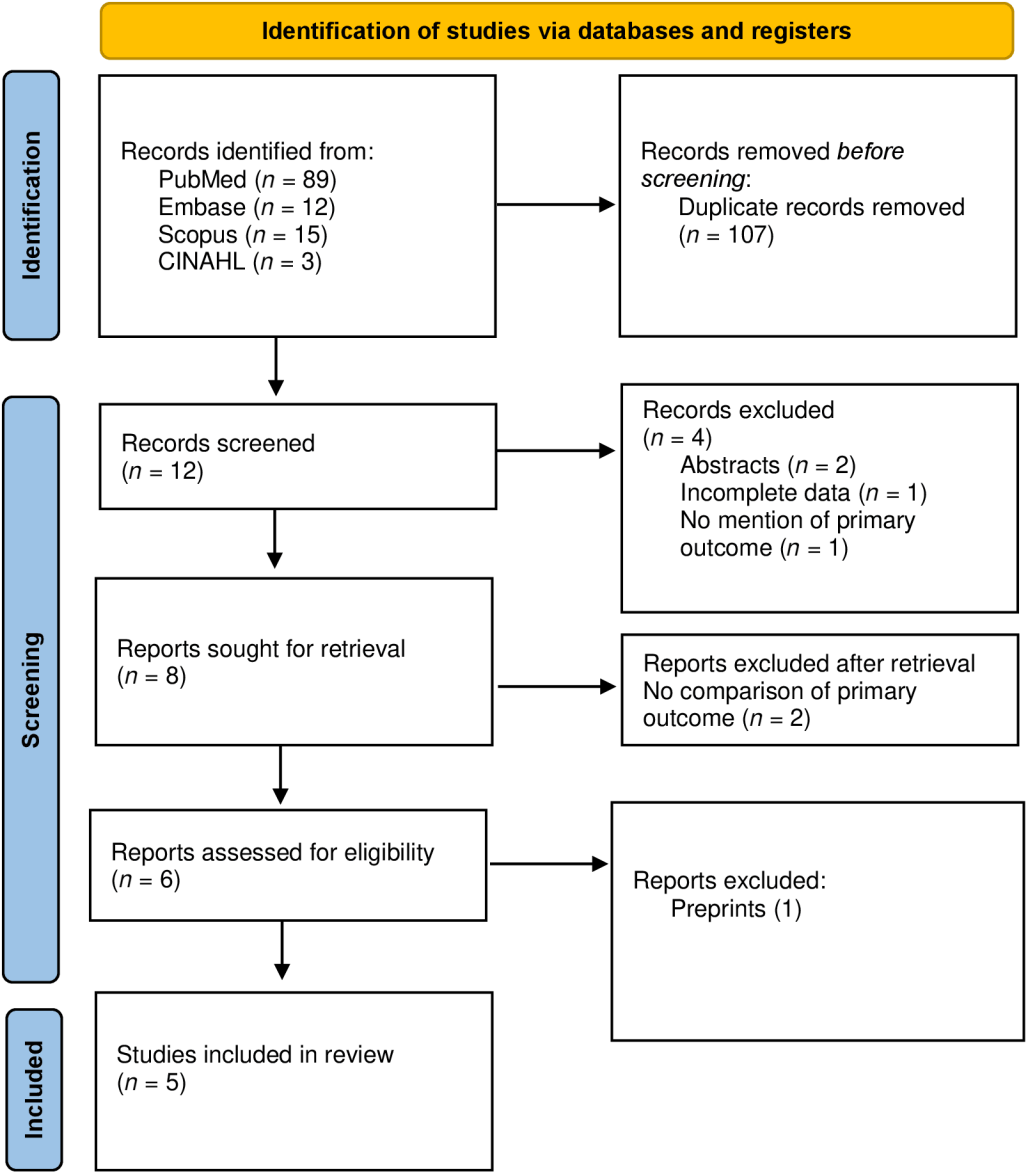
PRISMA flow chart.

**FIGURE 2:**
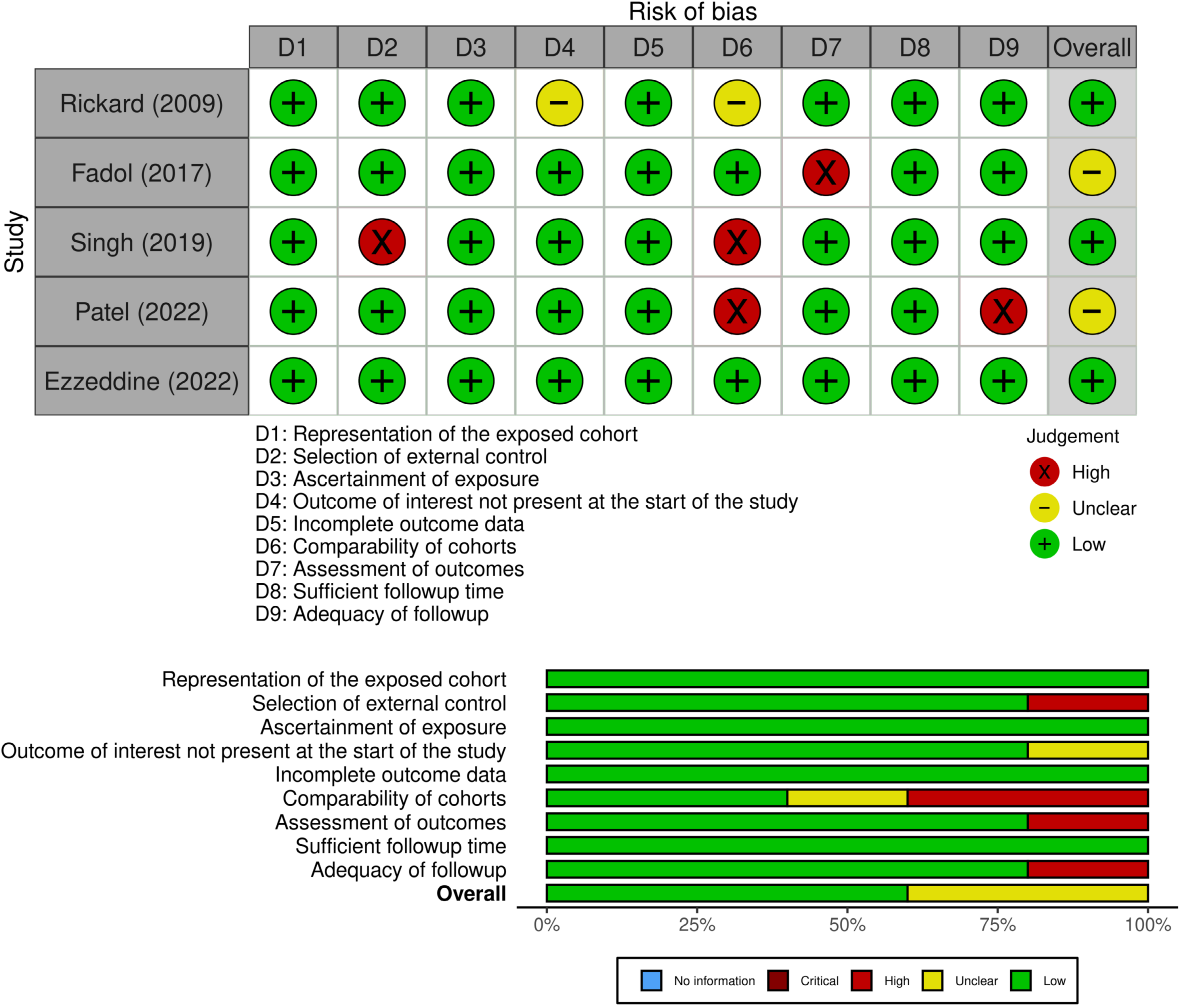
Study quality assessment.

A total of 119 articles were retrieved through the search strategy. After the removal of duplicates (67) and irrelevant articles (40), a total of 12 articles were sought for full text. Of these, 7 articles were excluded due to multiple reasons. Finally, a total of 5 articles were deemed eligible for review. PRISMA flow chart is shown in Figure 1 and the quality assessment is demonstrated in Figure 2.

### 3.2 Study characteristics

All five studies eligible for review were done in The United States of America and published between 2009 and 2022. Only one study was a prospective cohort while the remaining 4 studies were retrospective cohorts in nature. These 5 studies included a total of 169 patients who underwent CRT after CIC. Of these 169 patients, 61 (36.1%) patients were males. The mean age of the study participants was 62.9 ± 12.1 years. The majority of the patients were being treated for breast cancers, Hodgkin's lymphoma, soft tissue sarcomas, and acute lymphoblastic leukemias. All patients were given anthracyclines (doxorubicin or daunorubicin) as a part of chemotherapy protocols. These Baseline characteristics of the study participants are elucidated in Table 1.

**TABLE 1:** Baseline demographics and study characteristics.

Authors	Study year	Country	Study design	Sample size	Male (%)	Mean age	Type of cancers	Chemotherapy agents used
Rickard et al.	2009	United States	Retrospective cohort	18	2 (11%)	62.1 ± 11.1	Breast, non‐Hodgkin lymphoma, B‐cell lymphoma, acute lymphocytic leukemia	Doxorubicin
Fadol et al.	2017	United States	Retrospective cohort	58	31 (53.4%)	63.8% were more than 65 years	Breast cancer, sarcomas, acute lymphoblastic leukemias	Anthracyclines
Singh et al.	2019	United States	Prospective cohort	30	4 (13%)	64 ± 11 years	Breast cancer, Lymphoma, leukemias	Anthracyclines
Mezzedin et al.	2021	United States	Retrospective cohort	29	13 (44.8%)	66.3 ± 13.8	Lymphoma, leukemias	Doxorubicin
Patel et al.	2022	United States	Retrospective cohort	34	11 (32.3%)	60.5 ± 12.7	Lymphoma, leukemias	Anthracyclines

Abbreviations: AIC, Adriamycin‐induced cardiomyopathy; NIC, non‐ischemic cardiomyopathy.

### 3.3 Study outcomes

Rickard et al. reported an improved baseline LVEF from 18.6 ± 7.6% to 27.2 ± 13.5% and NYHA class improvement from 2.9 ± 0.3 to 2.4 ± 0.3 (Rickard et al., 2010). Fadol et al. (2017) only reported improved LVEF from 35% to more than 40% after CRT. Singh et al. (2019) reported improvement of LVEF from 28.5 ± 3.8% to 39.1 ± 7.1%, LVESV from 122.7 mL to 89 mL, LVEDV from 171 to 143.2 mL, and an improvement in NYHA class after CRT implantation. Ezzeddine et al. (2021) and Patel et al. (2022) demonstrated improved LVEF from 28 ± 8% to 38 ± 10% and 21.7 ± 7.4% to 30.4 ± 13.0%, respectively (Table 2).

**TABLE 2:** An elucidation of outcomes studied in the retrieved articles.

Authors	Baseline LVEF	Follow‐up LVEF	Baseline NYHA score	Follow‐up NYHA score	Outcomes
Rickard et al.	18.6 ± 7.6	27.2 ± 13.5	2.9 ± 0.3	2.4 ± 0.3	Patients with AIC demonstrate significant improvements in LVEF, LV end‐diastolic and end‐systolic diameters, mitral regurgitation, and NYHA functional class with CRT
Fadol et al.	≤35%	40.1 ± 5.7	Not reported	Not reported	CRT is underutilized in cancer patients with HF. CRT may be less utilized in those patients due to shortened life expectancy, yet evidence suggests that CRT has beneficial effects on morbidity, mortality, and left ventricular function
Singh et al.	28.5 ± 3.8	39.1 ± 7.1	NYHA II: 17/30 NYHA III: 13/30	NYHA II: 15/26 NYHA III: 11/26	CRT is associated with improvement in LVEF after 6 months The findings are limited by the small sample size, short follow‐up, and absence of a control group
Mezzedin et al.	28 ± 8	38 ± 10	Not documented	Not documented	CRT improves left ventricular function in terms of an increase in LVEF, and a decrease in LVEDD, and LVESD. CRT reverses remodeling in patients with CHIC
Patel et al.	21.7 ± 7.4	30.4 ± 13.0	Not reported	Not reported	Patients with anthracycline‐induced cardiomyopathy undergo LV remodeling with CRT at rates similar to other etiologies of NICM. Furthermore, AIC post‐CRT responders have favorable long‐term mortality compared to non‐responders

## 4 DISCUSSION

This systematic review represents the most contemporary data to demonstrate the efficacy of CRT in patients with CIC. It shows an improvement in LVEF from baseline along with other echocardiographic parameters. However, these investigations did not study other parameters of LV synchrony or QRS duration on ECG. Additionally, findings are limited by the small sample size, short follow‐up, and the absence of a control group. Despite these limited studies, a pool of evidence suggests a benefit of CRT implantation in this subset of heart failure patients.

Contemporary cancer treatments have significantly improved survival in solid‐organ tumors and a variety of hematological malignancies (Cardinale et al., 2015). With this treatment, however, a concomitant increase in heart failure is also observed secondary to the anti‐neoplastic properties of these drugs (Baech et al., 2018; Cardinale et al., 2015). The mainstay of treatment for a large number of malignancies is anthracyclines, which are known to cause cardiotoxicity and heart failure in a substantial proportion of patients (Baech et al., 2018). CIC, associated with anthracycline and other anti‐neoplastic drugs, is a well‐recognized entity, and many patients present with LV dysfunction in the first year of receiving the treatment (Singh et al., 2019). Investigations have suggested a 50% mortality rate with progressive heart failure due to chemotherapy compared with other nonischemic causes of heart failure (Goorin et al., 1981).

CRT is a well‐known guideline‐based therapy that can provide LV synchrony through electrical resynchronization (Sami et al., 2023). The first proof of concept for CRT in the CIC patient subset was provided in a retrospective analysis of 4 patients, which led to the design of the other prospective studies (Rickard et al., 2010). The studies included in this review show a similar improvement in LVEF as other large RCTs (MADIT‐CRT [10.6%] and MADIT‐CHIC [12.6%]) (Moss et al., 2009; Singh et al., 2019).

The specialty of cardio‐oncology provides multidisciplinary management of this growing patient subset. However, it is still limited by a lack of consolidated data and guidelines on device implantation and patient management. Although some evidence suggests that early initiation of anti‐heart failure medicines can reverse this condition, however, many patients with CIC and LV dyssynchrony do not respond to pharmacological therapy alone (Singh et al., 2019). The primary benefit of CRT in this patient subset can be due to the direct reversal of LV dyssynchrony and improved positive remodeling of the heart (Gulati et al., 2016).

This review had several limitations. First, the included studies had few patients and short‐term outcomes and follow‐ups. Second, there was a lack of control group paring CRT vs medical therapy of CRT in CIC vs other cardiomyopathies that did not allow estimation of potential effect size independent of spontaneous improvement or random variation of LVEF. Third, the criteria for diagnosis of CIC were only center‐standardized and there could be a chance of mislabeled diagnosis and management. Fourth, CRT parameters were not defined in the studies, and heart failure treatment was not shown in the studies.

## 5 CONCLUSION

In this systematic review, CRT was associated with improvement in all echocardiographic parameters, including LVEF, reduction in LV volumes, and NYHA class. The findings are limited by the small sample size, short follow‐up, and absence of a control group.

## AUTHOR CONTRIBUTIONS

Conceptualization: JM; First draft: MS, HK, HMS, BU, AZ; Methodology: AU, SMJZ, JB; Supervision: AM, JM; Final draft: AM, JM, SMJZ.

## CONFLICT OF INTEREST STATEMENT

The authors declare no competing interests.

## Data Availability

Data sharing is not applicable to this article as no new data were created or analyzed in this study.
